# Increased hallux angle in children and its association with insufficient length of footwear: A community based cross-sectional study

**DOI:** 10.1186/1471-2474-10-159

**Published:** 2009-12-17

**Authors:** Christian Klein, Elisabeth Groll-Knapp, Michael Kundi, Wieland Kinz

**Affiliations:** 1 Emco Clinic Bad Dürrnberg, Prof Martin Hell Str 7-9, 5422 Bad Dürrnberg, Austria; 2 Institute for Environmental Health, Center for Public Health, Medical University of Vienna, Kinderspitalgasse 15, 1090 Vienna, Austria

## Abstract

**Background:**

Wearing shoes of insufficient length during childhood has often been cited as leading to deformities of the foot, particularly to the development of hallux valgus disorders. Until now, these assumptions have not been confirmed through scientific research. This study aims to investigate whether this association can be statistically proven, and if children who wear shoes of insufficient length actually do have a higher risk of a more pronounced lateral deviation of the hallux.

**Methods:**

858 pre-school children were included in the study. The study sample was stratified by sex, urban/rural areas and Austrian province. The hallux angle and the length of the feet were recorded. The inside length of the children's footwear (indoor shoes worn in pre-school and outdoor shoes) were assessed. Personal data and different anthropometric measurements were taken. The risk of hallux valgus deviation was statistically tested by a stepwise logistic regression analysis and the relative risk (odds ratio) for a hallux angle ≥ 4 degrees was calculated.

**Results:**

Exact examinations of the hallux angle could be conducted on a total of 1,579 individual feet. Only 23.9% out of 1,579 feet presented a straight position of the great toe. The others were characterized by lateral deviations (valgus position) at different degrees, equalling 10 degrees or greater in 14.2% of the children's feet.

88.8% of 808 children examined wore indoor footwear that was of insufficient length, and 69.4% of 812 children wore outdoor shoes that were too short. A significant relationship was observed between the lengthwise fit of the shoes and the hallux angle: the shorter the shoe, the higher the value of the hallux angle. The relative risk (odds ratio) of a lateral hallux deviation of ≥ 4 degrees in children wearing shoes of insufficient length was significantly increased.

**Conclusions:**

There is a significant relationship between the hallux angle in children and footwear that is too short in length. The fact that the majority of the children examined were wearing shoes of insufficient length makes the issue particularly significant. Our results emphasize the importance of ensuring that children's footwear fits properly.

## Background

The etiology of hallux valgus deformities is complex. Besides intrinsic factors like heredity [1-6], pes planus [6,7], metatarsus primus varus [1,2,8], first metatarsal length [9-13] and a hypermobility of the metatarsocuneiform joint [1], extrinsic factors are also involved. Footwear - respectively the fit of shoes - seems to be a major extrinsic factor, since hallux valgus occurs preferential in shoe-wearing populations [5,14-16].

The fit of shoes is defined by numerous parameters such as length, width (ball and heel), girth, and height of toe box and shape. However, length seems to be one of the most important parameters [17]. In this paper, inside length of children's footwear was analysed (i.e. lengthwise fit).

The fit of children's footwear is often insufficient, with at least half of all children wearing shoes of insufficient length [18-21]. It has been indicated that this could lead to a deviation of the great toe [8,14,18,21-31].

There is general agreement in studies conducted over the past 50 years concerning adverse consequences of poorly fitting shoes on children's feet. Hallux valgus deformities are attributed to poorly-fitting footwear [14,21,25,27,29,32,33]. In addition, splay-foot and other deformities of the toes have been reported [19]. Eckstein and Schmidt report contractures in the forefoot area, visible in podogram images of the metatarsal capitulum, possibly a sign of forefoot damage [21,29]. Muscular damage and deformity of the forefoot attributed to the constant exposure of small forces, such as those exerted by shoes of insufficient length, were also observed [24]. In addition, damage to children's feet as a result of poorly-fitting shoes was sometimes reported, but without precise descriptions of the damage [17,22,27,28].

None of the studies to date, however, has investigated whether there is a significant relationship between the wearing of shoes of insufficient length and a lateral deviation of the great toe.

The objective of this study was to systematically investigate the relationship between insufficient length of footwear and the hallux angle in children, and to assess these children's risk of having a more pronounced lateral deviation of the great toe than children whose shoes fit properly. Further, for the first time, the study intends to differentiate between outdoor shoes and footwear worn inside the home or at child care facilities (indoor shoes), because children in Austria spend more time per day wearing indoor than outdoor shoes.

Pre-school children participated in the study. This study population was chosen in order to examine the fit of the children's footwear at an early age and to be able to observe possible negative effects at a juvenile developmental stage.

## Methods

### Study group, study site

Overall, 858 pre-school children, 439 boys and 419 girls (aged 3 to 6.5 years, 4.88 ± 0.029 SE) participated in the study. Sampling was stratified by gender, residential area (urban/rural) and different provinces corresponding to the Austrian population. The sample size was based on the following considerations: At least 50 children should be included in each of the 16 subgroups, defined by gender (2), rural vs. urban residential area (2) and province (4). This sample size guarantees a power above 90% to detect a moderate correlation, even in subgroups. Children were tested in pre-schools. Parental consent was a prerequisite for the children's participation. The drop-out rate was below 0.5%.

A carefully history and a static and dynamic clinical examination were conducted on the barefoot children. Data of children with clubfoot deformity, pes adductus, visible bunion deformities and surgical treatment of these deformities were excluded from statistical analysis (drop-out rate 1.15%).

The study was commissioned, approved and conducted by the Austrian Federal Ministry of Health (GZ 238.002/0-VIII/A6/02).

At the time the study was initiated and conducted (from 2001 to 2003), it was not mandatory under Austrian law for studies of this type to apply for approval from the ethics committee. However, all provisions of the Helsinki declaration concerning studies with human subjects were followed.

### Data collection, Anthropometric measures

3-D measurements were taken of the children's feet in an upright position (pedus 3-D Scanner, tecmath). Foot length was defined as the distance between the back of the heel and the tip of the longest toe. Measurements were precise to 0.01 mm. For ethical reasons, an assessment of the hallux angle based on x-ray measurements was not performed in this healthy population of children. The angle of the hallux was therefore measured on a foot outline as described by Barnicot & Hardy and Wülker [30,33]. According to Barnicot & Hardy, this external measurement corresponds closely to the radiographic measurements (r = 0.56) [30].

**Figure 1:**
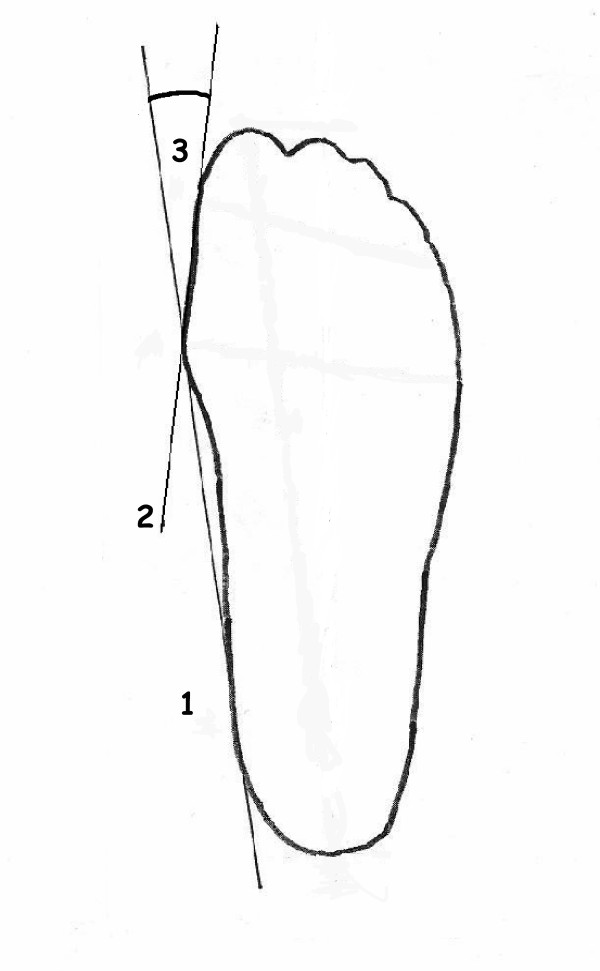
**Measuring the angle of the hallux**.

A straight line (1) was drawn through the most medial points of the first metatarso-phalangeal joint and the heel (inside edge). A second straight line (2) was drawn through the first metatarso-phalangeal joint and the great toe (proximal phalanx). According to the medial or lateral deviation of the great toe in relation to the inside edge, the angle between these two straight lines (3) was then recorded as the hallux angle in a valgus or varus position (Figure 1). Measurements were assessed in 1-degree steps. The interrater reliability of this method was r = 0.982, based on a sample of 90 footprints. All reported measurements were, however, conducted by only one person.

Due to the wide variation of cut-offs between normal and pathological angles found in the literature [19,31,33-37], we decided to assess the hallux angles with a precision of 1 degree using the method described above without a predefined cut-off point.

To our knowledge, no evidence-based normal values and ranges of the hallux angle are available for children of such a young age, either based on radiographic measurements or based on data assessed by the above described external method. Categories introduced in this study are based on classes of hallux angles (in degrees) without any reference to "normal" values.

The height and the weight of the children were measured and the BMI calculated according to the specific guidelines for young children [38,39].

### Data collection footwear

**Figure 2:**
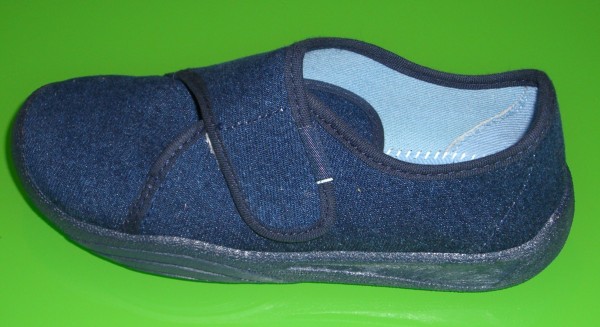
**Typical Austrian pre-school indoor shoe**.

The outdoor shoes that the children wore to pre-school on the days the data were collected and the indoor shoes they wore during the day were examined. Parents were not informed ahead of time on which days data would be collected. The typical indoor shoe for pre-school children in Austria is a commercial product with a thin outsole and a closed upper (Figure 2).

**Figure 3:**
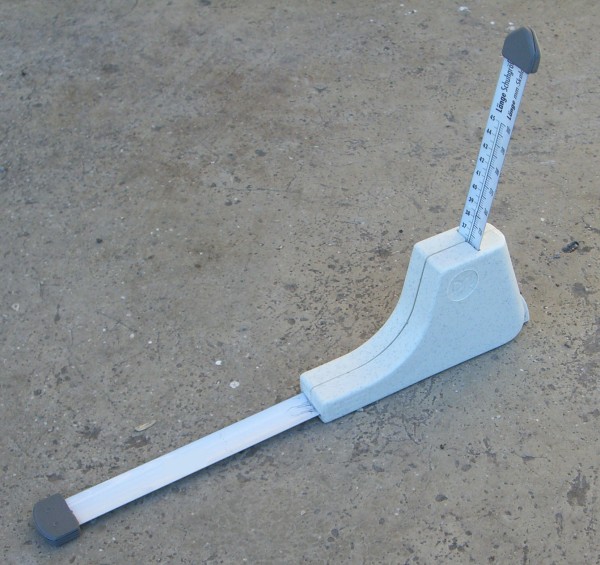
**Measuring device for the inside length of shoes**.

The inside length of the outdoor and indoor shoes was measured using an adapted sliding device designed for this purpose (Figure 3). To determine the inside length with a maximum level of precision, the device's scale, calibrated to show shoe size, was replaced with a commercially available measuring tape, precision class II. Measurements were conducted on the insole (back of the heel to the furthest point of the toe area) at one-millimetre intervals.

To calculate the lengthwise fit of the indoor and outdoor shoes, the difference between the actual inside length of the shoe and the length of the foot was determined with a precision of one millimetre. When classifying the results, it was taken into account that a properly fitting shoe should be at least 10 mm (optimally 12 mm) longer than the foot [17,28,29], and that the difference in length between shoe sizes is equal to 6.67 mm (EU Paris Point shoe sizes) (Table 1).

**Table 1:** Fit classification of shoes

Shoe minus foot	Fit classification (EU shoe size)
- 30.08 mm - (- 3.37) mm	More than 2 sizes too short
- 3.36 mm - (+ 3.31) mm	2 sizes too short
+ 3.32 mm - (+ 9.99) mm	1 size too short
+ 10 mm - (+ 16.67) mm	Correct fit
+ 16.68 mm - (+24 mm)	1 size longer and more

A properly-fitting shoe should be at least 10 mm and optimally 12 mm longer than the foot.

### Data analyses

Comparisons between boys and girls and left vs. right feet for hallux angle and for fit of shoes were done by Student's t-tests based on the respective numeric values. Categories for fit and categories for hallux angles were compared by Chi-square tests. Because left and right hallux angle had a highly significant correlation (Pearson correlation coefficient), and the hallux angle for left feet was more pronounced, only data for left feet were selected for analyses for fit criteria.

To test the functional association between hallux angle and the fit of shoes, a logistic regression analysis was performed with "fit" for street and indoor shoes as forced inclusion variables, and gender, age, body weight and body mass index as stepwise inclusion variables. For relative risk calculation, a cut off point of ≥ 4 degrees hallux angle was used. The cut-off was chosen based on the maximum of Nagelkerke's R^2 ^starting with one degree hallux angle and increasing the angle by one degree at each step.

## Results

### Hallux angle

**Figure 4:**
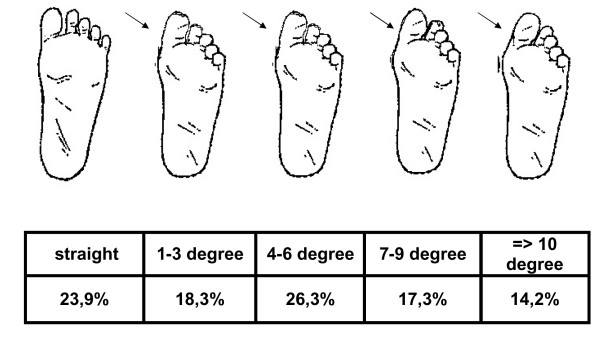
**Number of children in the 5 categories of hallux valgus angles**.

The hallux angle was determined precisely on a total of 1,579 feet (790 left feet, 789 right feet). The position of the great toe varied between a straight position and a hallux angle of up to 19 degrees valgus position. For demonstration purposes and for selected statistical analyses, the observed hallux angles were classified into 5 categories as presented in Figure 4. Only 23.9% of the children's feet presented a straight position of the great toe. In 18.3% of cases, the observed hallux angle ranged between 1 and 3 degrees valgus position, in 26.3% between 4 and 6 degrees and in 17.3% between 7 and 9 degrees. In 14.2% of the children's feet, a valgus deviation of equal to or greater than 10 degrees was observed.

The hallux valgus angle of the left and right feet were significantly correlated (r = 0.49, n = 784, p ≤ 0.001). However, the left hallux angles were on average 0.5 degrees greater (t = 1.55, df = 783, p = 0.12).

Valgus positions of the great toe were significantly more frequent in male than in female participants (chi^2 ^= 11.590, df = 1, p = 0.001). The hallux angle was greater in male than in female participants (t = 3.569, df = 788, p ≤ 0.001).

### Fit of shoes

Since there was no difference between the fit of right and left shoes, and due to the slightly worse hallux angle of the left feet, only data from left shoes were used for fit analyses. Only 22.8% of the 812 children wore properly-fitting outdoor shoes. The outdoor shoes of 69.4% of the children tested were too short in length. In the case of indoor shoes, the results were even worse. Only 9.4% of the 808 children wore properly-fitting indoor footwear, 88.8% of the children had indoor shoes of insufficient length. The frequency of the different categories of fit is shown in Table 2.

**Table 2:** Results of fit of outdoor and indoor shoes

	Outdoor shoes		Indoor shoes	
	n (children)	%	n (children)	%
More than 2 sizes too short	77	9.5	192	23.8
2 sizes too short	223	27.5	299	37
1 size too short	263	32.4	226	28
Correct fit	185	22.8	76	9.4
1 size longer and more	64	7.9	15	1.9

Total	812	100	808	100

Only 22.8% of the children wore properly-fitting outdoor shoes.

Only 9.4% of the children wore properly-fitting indoor shoes.

An analysis of gender effect in these categories of fit showed that boys were more likely to wear poorly-fitting shoes than girls. This applies to both, outdoor and indoor shoes (chi^2^_(outdoor shoes) _= 8.934, df = 4, p = 0.063; chi^2^_(indoor shoes) _= 10.89, df = 4, p = 0.028).

### Risk of hallux valgus deviation

The hallux angle (numeric values of the angle) and the lengthwise fit of the shoes (numeric values of the discrepancy between length of shoes and length of foot) show a clear relationship (r_(indoor shoes) _= -0.10, n = 763, p < 0.003; r_(outdoor shoes) _= -0.10, n = 767, p < 0.007): The shorter the shoe, the greater the hallux valgus angle.

A stepwise logistic regression analysis revealed a significant effect of the criterion lengthwise fit of indoor shoes on the risk of a more pronounced lateral deviation of the great toe. Effects of the criterion lengthwise fit of outdoor shoes showed a similar tendency, but were not significant. Of all variables included stepwise in the analyses, only the variable gender proved significant. Age, body weight, and body mass index had no significant effect. Interaction between gender and fit was tested and found to be not significant (p > 0.05).

The relative risk (odds ratio) for a hallux valgus angle of ≥ 4 degrees is 1.171 (95% CI 1.013-1.358), p = 0.03) if indoor shoes are one shoe size too short.

The relative risk (odds ratio) for a hallux valgus angle of ≥ 4 degrees is 1.048 (95% CI 0.916-1.202), p > 0.05) if outdoor shoes are one shoe size too short.

Hence the risk for a hallux valgus angle of ≥ 4 degrees is 17% higher than the background risk if indoor shoes are 1 size too short (1 EU size: 6.67 mm). This risk increases to 37% if shoes are 2 sizes too short, and to 61% for shoes 3 sizes too short.

The non-significantly increased odds ratio for outdoor shoes corresponds to a risk increase of 5% for children wearing shoes which are 1 size too short. For shoes which are 2 sizes too short it increases to 10% and to 15% for shoes which are 3 sizes too short.

Male gender was associated with an odds ratio of 1.573 (95% CI 1.169-2.116, p = 0.003). For boys, the relative risk was 57% higher than for girls, independent of possible differences in the absolute fit of shoes between boys and girls.

## Discussion

In order to assess the relationship between poorly-fitting shoes and risk of hallux valgus, we choose a representative cross-sectional study design. A significant correlation between the fit of shoes and the hallux valgus angle and an increased risk for hallux angles ≥ 4 degrees was found, indicating poor fit, particularly in indoor shoes, as a significant factor contributing to podiatric pathology.

Although it has been speculated upon frequently in the literature [8,14,18,21-31], this investigation provides the first systematic empirical data supporting the assumption that shoes of insufficient length are a risk for healthy foot development. This study was also the first to examine children of this young age before school entry (3 - 6.5 years of age).

Furthermore, data published to date provide no information about indoor shoes. According to our results, only few children wear indoor shoes that fit well, and the fit of indoor shoes is generally poorer than that of outdoor shoes. This may partly be due to the fact that parents pay less attention to indoor shoes than to outdoor shoes. Pre-school teachers reported that in many cases, the indoor shoes were not replaced for a whole school year. The risk of a greater hallux angle for children wearing poorly-fitting indoor shoes is markedly higher than for children wearing poorly-fitting outdoor shoes. The increased risk might be due to the worse fit of the indoor shoes, and to the fact that children of this age group spend more time per day wearing indoor than outdoor shoes.

Considering the fact that about 61% of the children in our investigation wore indoor shoes 2 sizes too short, associated with an increase of 37% for the risk of a hallux angle of 4 degrees or more, the issue is of considerable practical importance.

There are, however, some limitations to our study.

Intrinsic risk factors for an increase in the hallux angle were only partly controlled in our study as no x-ray images were available for this healthy childhood population. But children's feet were examined for clinical signs during a static and dynamic clinical examination, and data from children with clinical signs were excluded from analyses. No information about hallux valgus family history was available. However, it seems unlikely that children with a family history of hallux valgus would wear shoes of insufficient length more frequently than others, increasing the association between too-short shoes and increased hallux angle found in our study.

Hallux angle assessment is performed by radiographic measurements in most studies. In our study, for ethical reasons, an external measurement method based on footprints was used. Barnicot & Hardy [30] found that the hallux angle measured on footprints in the manner mentioned above showed a correlation of r = 0.56 with measurements of the angle between the first toe and the metatarsal made on radiographs of the same sample. They conclude that despite the high correlation, caution is required in inferences made from data obtained by one method to data obtained by the other. The same, however, holds true even for different radiographic methods for hallux angle assessment, as was shown by Schneider & Knahr [40].

Since we assessed shoes at the same time as foot measurements were conducted, we were not able to investigate the relationship between the duration of wearing poorly-fitting shoes and the development of pathologies. This results in exposure misclassification, reducing the risk estimates, because children with appropriate shoes might have purchased them recently and worn them for just a short period of time. But it is unlikely that non-fitting shoes were worn only on the particular day when measurements were done. Another important aspect that should be addressed in future investigations is whether, after changing footwear habits, hallux valgus is reversible and how long it takes for the changes to develop. Eckstein [21] and Craigmile [13] provide initial, encouraging findings on this question in studies in which a reversal was observed in some children after periods of wearing properly-fitting shoes.

In the meantime, a new research project in cooperation with the Austrian Ministry of Health has been started. It is dedicated to finding out what has to be done to ensure that children have correctly fitting shoes, and to prove the effects of intervention programmes.

## Conclusions

It could be proven that the risk of having a hallux angle deviation is increased in children wearing shoes of insufficient length. The fact that 88.8% of the children examined wear indoor shoes of insufficient length, and 69,4% wear outdoor shoes which are too short, points to the general health relevance of this problem.

From a public health perspective, these findings are especially important in light of the large number of children wearing poorly-fitting shoes. Therefore, it is necessary to provide parents and the general public with comprehensive information on the importance of properly-fitting shoes and the criteria of a proper fit.

## Competing interests

The authors declare that they have no competing interests.

## Authors' contributions

EGK was responsible for the overall design of the study. Acquisition of data: CK and WK. Analysis and interpretation of data: EGK. Biostatistics: MK. All authors helped to draft the manuscript, read and approved the final manuscript.

## Pre-publication history

The pre-publication history for this paper can be accessed here:

http://www.biomedcentral.com/1471-2474/10/159/prepub

## References

[B1] Coughlin MJ Mann RA Sugery of the Foot and Ankle 1999 1 7 Philadelphia: Mosby 151 168

[B2] Hardy RH Clapham JCR Observations on Hallux valgus J Bone Joint Surg [Br] 1951 33-B 376 391 14861244 10.1302/0301-620X.33B3.376

[B3] Glynn MK Dunlop JB Fitzpatrick D The Mitchell distal metatarsal osteotomy for Hallux valgus J Bone Joint Surg [Br] 1980 62-B 188 191 7364833 10.1302/0301-620X.62B2.7364833

[B4] Johnston O Further studies of the inheritance of hand and foot anomalies Clin Orthop 1956 8 146 160 13374909

[B5] Wanievenhaus A Bock P Gruber F Ivanic G Klein C Siorpaes R Schneider W Steinböck G Trieb K Trnka HJ Deformity associated treatment of the hallux valgus complex Orthopade 2009 in press 10.1007/s00132-009-1526-3 19730810

[B6] Coughlin MJ Jones CP Hallux Valgus: demographics, etiology and radiographic assessment Foot Ankle Int 2007 28 759 777 10.3113/FAI.2007.0759 17666168

[B7] Mann RA Coughlin MJ Hallux valgus: etiology, anatomy treatment and surgical considerations Clin Orthop 1981 157 31 7249460

[B8] Coughlin MJ Juvenil hallux valgus: etiology and treatment Foot Ankle Int 1995 16 682 697 8589807 10.1177/107110079501601104

[B9] Harris RI Beath T The short first metatarsal: its incidence and clinical significance J Bone Joint Surg [Am] 1949 31-Am 553 565 18146111

[B10] Morton DJ The human Foot 1935 New York: Columbia University Press

[B11] Wheeler Haines R McDougall A The anatomy of hallux valgus J Bone Joint Surg [Br] 1954 36-B 272 293 13163115 10.1302/0301-620X.36B2.272

[B12] Mayo CH The surgical treatment of bunions Minn Med J 1920 3 326 331

[B13] Craigmile Doris A Incidence, origin and prevention of certain foot defects Br Med J 1953 2 749 752 10.1136/bmj.2.4839.749 13082099 PMC2029547

[B14] Sim-Fook L Hodgson AR A comparison of foot forms among the non-shoe and shoe-wearing Chinese population J Bone Joint Surg [Am] 1958 40 1058 1062 13587573

[B15] Coughlin MJ Hallux valgus Causes, evaluation and treatment Postgrad Med 1984 75 174 187 6709527 10.1080/00325481.1984.11698000

[B16] Menz HB Morris ME Footwear characteristics and foot problems in older people Gerontology 2005 51 346 351 10.1159/000086373 16110238

[B17] Cheng FT Perng DB A systematic approach for developing a foot size information system for shoe last design Int J Ind Ergon 1999 25 171 185 10.1016/S0169-8141(98)00098-5

[B18] Timm H Measurement of the length of children's shoes with shoe-measurement cards Z Orthop Ihre Grenzgeb 1963 97 384 386 14045169

[B19] Debrunner HU Wachstum und Entwicklung des Fußbes beim Jugendlichen (Growth and development of the juvenile foot) Supplement to no. 99 of the Z Orthop 1965 Stuttgart: Ferdinand Enke Verlag

[B20] Bauer R Frischhut B Der Vorfuß Orthopade 1996 25 301 10.1007/s001320050028 8927374

[B21] Eckstein G Der nicht passende Schuh und das Problem erworbener Vorfußschäden bei Kindern (Poorly-fitting shoes and the problem of acquired forefoot damage) PhD thesis 1990 Erlangen-Nürnberg

[B22] Maier E Killmann M Kinderfuß und Kinderschuh. Entwicklung der kindlichen Beine und Füß und ihre Anforderungen an fußgerechte Schuhe 2003 München: Verlag Neuer Merkur

[B23] Kato T Watanabe S The etiology of hallux valgus in Japan Clin Orthop Relat Res 1981 157 78 81 7249466

[B24] Stracker O Child's foot and child's shoe Arch orthop Unfallchir 1966 59 286 294 10.1007/BF00415251 5996423

[B25] Kristen H The child's foot in Austria Z Orthop Ihre Grenzgeb 1968 3 318 333 4233339

[B26] Hellbruegge T Zur Prophylaxe erworbener Fußschäden. Kinderschuh-Seminare für Kinderärzte (Preventing acquired foot damage. Seminars on children's footwear for pediatricians) Sozialpädiatrie in Praxis und Klinik 1980 3

[B27] Maier E Erworbene Fußschäden bei Kindern und Jugendlichen (Acquired foot damage of childrens and juveniles) Sozialpädiatrie in Praxis und Klinik 1982 6 297 307

[B28] Dhom G Das Zugabeproblem bei fußgerechten Kinderschuhen (The problem of extra length in correctly fitting children's shoes) PhD thesis 1984 Johannes Gutenberg Universität Mainz

[B29] Schmidt C Die optimale Längenzugabe in Sport- und Freizeitschuhen bei Kindern (The optimal length of sport shoes and recreational footwear for children) PhD thesis 1989 Erlangen-Nürnberg

[B30] Barnicot NA Hardy RH The position of the hallux in West Africans Am J Anat 1955 89 355 361 PMC1244763 13251965

[B31] Pisani G Fußchirurgie 1988 Georg Thieme Verlag Stuttgart

[B32] Shine IB Incidence of hallux valgus in a partially shoe-wearing community Br Med J 1965 1 1648 1650 10.1136/bmj.1.5451.1648 14298843 PMC2167058

[B33] Wülker N Hallux valgus-hallux rigidus 1997 Stuttgart: Ferdinand Enke Verlag

[B34] Jerosch J Mamsch H Deformities and misalignment of feet in children - a field study of 345 students Z Orthop Ihre Grenzgeb 1998 136 215 220 10.1055/s-2008-1054225 9736981

[B35] Forst R Venbrocks R, Salis-Soglio G Spreizfuß und Hallux valgus des Jugendlichen (Juvenile splay foot and hallux valgus) Jahrbuch der Orthopädie 1993 Zülpich: Biermann 63 72

[B36] Schilling W Morphologische und funktionelle Entwicklung des Kinderfußes (Morphological and functional development of children's feet) Medizinische Orthopädietechnik 1982 109 2 6

[B37] Kilmartin TE Barrington RL Wallace WA Metatarsus Primus Varus. A statistical study J Bone Joint Surg [Br] 1991 73-B 937 940 10.1302/0301-620X.73B6.1955440 1955440

[B38] Krohmeyer-Hauschild K Wabitsch M Kunze D Geller F Geiß HC Hesse V von Hippel A Jaeger U Johnsen D Korte W Menner K Müller G Müller JM Niemann-Pilatus A Remer T Schaefer F Wittchen HU Zabransky S Zellner K Ziegler A Hebebrand J Perzentile für den Body-mass Index für das Kindes- und Jugendalter unter Heranziehung verschiedener deutscher Stichproben (Percentiles of body mass index in children and adults evaluated from different regional German studies) Monatsschr Kinderheilkd 2001 149 807 818 10.1007/s001120170107

[B39] AGA (Arbeitsgemeinschaft Adipositas im Kindes- und Jugendalter) http://www.a-g-a.de/aga_content.html

[B40] Schneider W Knahr K Metatarsophalangeal and Intermetatarsal Angle: Different Values and Interpretation of Postoperative Results Dependent on the Technique of Measurements Foot and Ankle Int 1998 19 532 536 10.1177/107110079801900805 9728700

